# Organochlorine pesticides and epigenetic alterations in thyroid tumors

**DOI:** 10.3389/fendo.2023.1130794

**Published:** 2023-07-25

**Authors:** Fouzieh Salimi, Gholamreza Asadikaram, Mohammad Reza Ashrafi, Hamid Zeynali Nejad, Moslem Abolhassani, Mojtaba Abbasi-Jorjandi, Mojgan Sanjari

**Affiliations:** ^1^Endocrinology and Metabolism Research Center, Institute of Basic and Clinical Physiology Sciences Kerman University of Medical Sciences, Kerman, Iran; ^2^Applied Cellular and Molecular Research Center, Kerman University of Medical Sciences, Kerman, Iran; ^3^Department of Clinical Biochemistry, School of Medicine, Kerman University of Medical sciences, Kerman, Iran; ^4^Physiology Research Center, Institute of Neuropharmacology, Kerman University of Medical Sciences, Kerman, Iran; ^5^Department of Surgery, School of Medicine, Kerman University of Medical Sciences, Kerman, Iran

**Keywords:** thyroid tumors, DNA methylation, histone modification, organochlorine pesticides, epigenetic alterations

## Abstract

**Purpose:**

Cancer incidence depends on various factors e.g., pesticide exposures which cause epigenetic alterations. The present research aimed to investigate the organochlorine pesticides (OCPs) impacts on promoter methylation of three tumor-suppressor genes and four histone modifications in thyroid nodules in 61 Papillary thyroid carcinoma (PTC) and 70 benign thyroid nodules (BTN) patients.

**Methods:**

OCPs were measured by Gas chromatography. To identify promoter methylation of TSHR, ATM, and P16 genes, the nested-methylation-specific PCR (MSP) was utilized, and histone lysine acetylation (H3K9, H4K16, and H3K18) and lysine methylation (H4K20) were detected by performing western blot analysis.

**Results:**

Further TSHR methylation and less P16 methylation were observed in PTC than in BTN. No substantial difference was detected for ATM methylation between PTC and BTN groups. Also, OCP dramatically increased the odds ratio of TSHR (OR=3.98, *P*=0.001) and P16 (OR=5.65, *P*<0.001) methylation while confounding variables reduced the chances of ATM methylation arising from 2,4-DDE and 4,4-DDT influence. Hypomethylation of H4K20 and hypo-acetylation of H3K9, H4K16, and H3K18 (*P*<0.001) were observed in PTC samples than BTN. Furthermore, OCPs substantially decreased the odds ratio of H3K9 (OR=3.68, *P*<0.001) and H4K16 (OR=6.03, *P*<0.001) acetylation.

**Conclusion:**

The current research indicated that OCPs could contribute to PTC progression by TSHR promoter hypermethylation and decreased acetylation of H3K9 and H4K16. In addition, in PTC patients, assessing TSHR promoter methylation and acetylation of H3K9 and H4K16 could have predictive values.

## 1 Introduction

The most prevalent histological subtype of thyroid carcinoma is papillary thyroid cancer (PTC) (1). PTC includes more than 90% of thyroid carcinomas, and in recent years, it has quickly expanded (1, 2). The precise causes of PTC development are unknown, but some studies reported that environmental, epigenetic, and genetic factors could contribute to PTC progression (3, 4). Environmental agents, including bisphenol A (BPA), pesticides, and UV, can exert their influences through epigenetic mechanisms, such as DNA methylation and histone modifications, which regulate gene expression (5–8).

Aberrant DNA methylation at the CpG regions can abnormally silence gene expression and cause carcinoma progression (9, 10). Studies on human beings have shown the correlation of promoter hypermethylation of thyroid-stimulating hormone receptor (TSHR), ataxia telangiectasia mutated (ATM), and cyclin-dependent kinase inhibitor 2A (P16) with 34–59% (11, 12), 50% (11), and 27-35.9% (13–15) of the PTC cases, respectively (12). TSHR is a surface glycoprotein receptor, part of the leucine-rich repeat subfamily of G-protein-coupled receptors (LGR). It is crucial in regulating thyroid growth, differentiation, and thyroid hormone secretion (16). Since iodine metabolism depends on TSHR, researchers think that aberrant methylation in promoter regions of TSHR may contribute to thyroid cancer development (17). ATM gene produces a serine-threonine protein kinase, which activates checkpoint signaling against DNA double-strand break (DSB) (18). Therefore, unsurprisingly, previous studies have shown that it is aberrantly methylated in various cancers, such as breast (19), colorectal (20), hepatocellular carcinoma (21), and PTC (12). Regulation of the cell proliferation process is carried out by the P16 protein by inhibiting the G1/S phase transition in the cell cycle. Reduced expression of the P16 gene arising from its aberrant promoter methylation could substantially increase PTC risk (3). Exposure to high doses of polychlorinated biphenyls decreases CpG islands’ methylation in the P16 gene promoter (22).

Nuclear globular proteins known as histones can be covalently altered to affect chromatin structure and gene expression. These modifications include acetylation (Ac), methylation, phosphorylation, glycosylation, sumoylation, ubiquitination, and ADP ribosylation (23). All DNA-based processes, such as chromatin compaction, nucleosome dynamics, transcription, DNA repair, reproduction, stemness, and alterations in the cellular state, are affected by histone modification (8). These processes are commonly dysregulated in human cancers because they can alter the balance of gene expression (24). Adenomas and carcinomas had higher levels of Ac of histone 3 (H3) lysine residues 9-14 (H3K9-14ac) than usual, and lower than normal levels of H3 lysine 18 acetylation (H3K18ac) when the global pattern of histone modifications in several thyroid tumor histotypes was examined (25). Chemical contaminants, including pesticides, could influence histone alterations or enzyme function, which modify histones (26). In mouse models, long-time exposure to dieldrin stimulated dopaminergic neuron degeneration in the striatum and substantia nigra arising from histone hyper-acetylation (27).

According to the results of *in vitro* research, carcinogenesis resulting from pesticides could be mediated by epigenetic alterations (28, 29). Several endocrine-disrupting pesticides, which are artificial substances, can mimic natural hormone role and perturb epigenetic changes (30). The endocrine organ’s function could be impacted by endocrine-disrupting chemicals (EDCs), which are inorganic or natural substances. Methoxychlor (MXC), which has been isolated from 1,1,1-trichloro-2,2-bis (4-methoxyphenyl) ethane (DDT), is one of the synthetic EDCs that can exert its transgenerational effects by DNA methylation and also other epigenetic alterations (31). Accumulating data suggest that DNA methylation and histone alterations examination in cancer are possibly vital parameters for etiology, biomarkers recognition, and progression of disease determination. The purpose of the present research was to investigate some OCPs (e.g., α-HCH, β-HCH, γ-HCH, 2,4 DDE, 4,4 DDE, 2,4DDT and 4,4DDT) impacts on the promoter methylation of TSHR, ATM, and P16 tumor suppressor genes and histone lysine acetylation (H3K18, H3K9, and H4K16) and lysine methylation (H4K20) in thyroid tumor patients.

## 2 Materials and methods

### 2.1 Subjects

In total, 131 human specimens were collected from thyroid nodules that were resected and snap-frozen during surgery, and then instantly kept at -80°C.

Specimens included 61 patients recently recognized with papillary thyroid carcinomas (PTC) and 70 patients with benign thyroid nodules (BTN) at Bahonar Hospital and Nimeh-Shaban Clinic, Kerman, Iran (July 2017-May 2019). An endocrine surgery specialist and a pathologist diagnosed PTC and BTN tumors. In this research, the exclusion requirements were ≤18 years of age, the dissatisfaction of patients, previous ionizing radiation exposure, chronic and autoimmune conditions history, consumption of vitamins or other supplements, the intake of alcohol, and hormone therapy. Also, Helsinki –based protocols were utilized by medics and patients. All participants signed written informed consent. The research was accepted by the ethics board of Kerman University of Medical Sciences (Code No.: IR. KMU. REC.1398.370).

### 2.2 Evaluation of OCP residues

Standards of OCP components, their derivatives, and internal standard (4,4-dichlorobenzophenone, DBP) were obtained from the Germany-based company of Ehrenstorfer. N-hexane, anhydrous sodium sulfate, and ethyl acetate were purchased from Merck (Germany), and sulphuric acid was obtained from Scharlab in Spain. An altered technique has been previously explained to evaluate OCP levels (28, 32–34). First of all, DBP was combined with 0.5 mL of serum. Second, 2 mL of n-hexane was utilized for sample extraction (2 times). Third, to isolate the organic portion of the resulting mixture, 200 µL of concentrated sulphuric acid was applied. The organic component was then dehydrated by 100 mg of anhydrous sodium sulfate. After that, the organic layer was centrifuged, and at room temperature, wholly dried. Ultimately, the extracted OCPs were dissolved in 100 µL of ethyl acetate. All specimens were injected into a gas chromatograph (GC) (Agilent 7890A, USA) to measure and detect OCPs.

### 2.3 DNA extraction

By using the phenol-chloroform method, the DNA was refined (35). To extract DNA, 50 mg of tissue specimen was used. To summarize, a buffer (50 mM Tris, 5 mM EDTA, pH 8, and 300 μg/ml proteinase K) was utilized for tissue digestion, and lysed cells were kept overnight at 56°C. At the 50 μl Tris-EDTA (TE) buffer (100 mM Tris-HCl pH 8.0, 10 mM EDTA pH 8.0), the purified DNA was solved and retained in -20°C. To investigate DNA quality, a Nanodrop spectrophotometer (ND-1000; Nanodrop Technologies; Thermo Fisher Scientific, Inc., Wilmington, DE) was used.

### 2.4 Bisulfite modification and methylation-specific PCR

According to the Tiwari et al. technique, DNA was treated with bisulfite (36). Using MSP, which has enough sensitivity to determine a single methylated allele among 1000 unmethylated ones, DNA methylation was assessed (37). **Table S1** indicates the sequence of primers derived from other papers (38–40). First, to denature DNA, 1 µg of genomic DNA was incubated in 2 M NaOH for 15 minutes at 50 °C. Second, an equal volume of 2 percent low-melting agarose was applied. Next, to create beads, 100 μL of cold mineral oil was added to the resulting mixture. After that, specimens were well combined with 500 μL of the solution of hydroquinone (Merck, Germany) and sodium bisulfite (Merck, Germany) and then kept at 55°C for 4 hours in the dark. Finally, the beads were transferred to a new microtube and washed with 1× TE buffer followed by de-sulfonation in 0.5M NaOH (2 times×15 min), 1× TE buffer, and 1mL of ddH2O (2 times×15 min). The two-stage nesting method was utilized to improve the technique ‘s precision (41). Hence, nested-MSP is one of the common approaches used for studying DNA methylation. We performed a nested, two-stage PCR approach. The stage-1 primers recognize the bisulfite-modified template but do not discriminate between methylated and unmethylated alleles. The stage-1 PCR products were diluted 50-fold, and specific primers for methylated or unmethylated template were used in a stage-2 PCR (**Table S1**). Image J software was used to quantify DNA methylation. Positive results are obtained when methylation exceeds non-methylation, and vice versa. Software determined each band’s density and compared it to the others. The comparison of two groups was completed lastly. The number of CG sites We evaluated was 3 for TSHR, 6 for ATM, and 7 for P16.

### 2.5 Histone extraction

Based on the approach mentioned earlier, the extraction of histone proteins was performed (38). In brief, sample homogenization was done in the TEB buffer (0.5% Triton X 100, and 2mM phenylmethylsulfonyl fluoride in PBS), and 200 mg/mL of a protease inhibitor cocktail was added. The mixture was sonicated 6 times (10 s each), and the supernatant was discarded after centrifugation. Next, three volumes of extraction buffer (0.5 N HCl + 10% glycerol) was added to pellets and held on ice for 30 minutes. After the centrifugation of the mixture (12,000 rpm for 5 minutes), 8 volumes of 12% trichloroacetic acid (TCA) were utilized to precipitate the supernatant and then kept overnight at -20°C.

Finally, centrifuged specimens (12,000 rpm for 5 minutes) were air-dried, diluted in distilled water, quantified by the Bradford method, and reserved at -80°C for use in Western blots.

### 2.6 SDS-PAGE and western blot analysis

15% sodium dodecyl sulfate-polyacrylamide gels (SDS-PAGE) were used to isolate proteins (final concentration 40 µg/lane). Next, isolated proteins were transferred to a polyvinylidene difluoride (PVDF) membrane and investigated with primary Abs overnight at 4°C at the defined concentrations; Proteins were assessed by the primary antibodies of H3K9ac, 1:1000 (#ab10812; Abcam, Cambridge, UK); H3K18ac, 1:1000 (#ab1191; Abcam); H4K20me3, 1:1000 (#ab9053; Abcam); H4K16ac, 1:1000 (#ab205718; Abcam), and also as control was employed H3 (#ab1791; Abcam). The membranes were washed 3 times with TBS/0.1% Tween 20 (15 min each) and then for 2 hours incubated with horseradish peroxidase-conjugated goat anti-rabbit secondary antibodies (Sigma). Enhanced chemiluminescence (ECL) Western blot analysis was used to visualize the bands of protein, and blots were exposed to X-ray film. Image J software was used to quantify histone marks.

### 2.7 Statistical analysis

The statistical analyses have been carried out by SPSS software ver. 22.0 (SPSS Inc., Chicago, IL), and statistically significant was presented as a *p*-value lower than 0.05. The relationship between gene methylation and OCPs was assessed by the logistic regression model. Moreover, this study conducted the quantitative estimation of the two variables association (42). The continuous and discontinuous parameters were demonstrated as mean ± standard error of the mean (mean ± SEM) and numbers (percentages), respectively. The Kolmogorov-Smirnov test was applied to examine the normality of the results. In order to compare the quantitative and qualitative data between the PTC and BTN groups, the independent sample t-test/Mann-Whitney U test and χ^2^/Fisher’s exact tests were used, respectively. To evaluate the potential cutoff sensitivity-specificity profile, receiver operating characteristic (ROC) curves and areas under the curves (AUC) have been measured.

## 3 Results

### 3.1 Demographic and clinical characteristics

Clinical pathological details, e.g., age, sex, size of the tumor, local metastasis of lymph nodes and distant, and the clinical stage were gathered and listed in **Table S2**.

As mentioned before, 61 patients with PTC and 70 patients with BTN were included in this research. DNA methylation and histone modifications were analyzed to determine epigenetic variations correlated with thyroid nodules. There was a significant difference in mean age between PTC and BTN groups (40 and 47 years old, respectively). Females comprised 80% of the BTN group and 75.4% of the PTC group, while 24.6% of participants in the PTC group and 20% of BTN patients were male. In total, 10.4% of the cases had smoking habits.

### 3.2 Organochlorine pesticides

**Table S3** compares the mean levels of OCPs in the serum of patients with PTC and BTN. The results exhibited no significant difference between the PTC and BTN groups.

### 3.3 The three tumor-suppressor genes methylation in thyroid tumors

**Figure 1:**
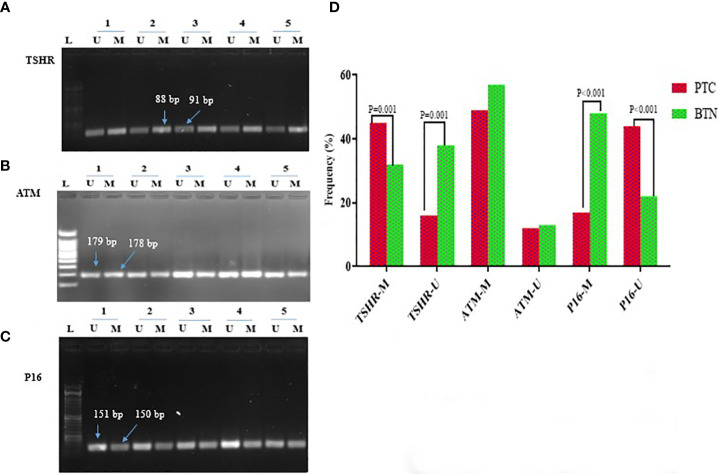
Using methylated specific PCR (MSP), five tumour samples were examined for the promoter methylation status of **(A)** TSHR, **(B)** ATM, and **(C)** P16, as well as **(D)** a chart that shows the differences between the groups. L = 100 bp DNA marker ladder, U = unmethylated and M = methylated promoter sequences.

**Figure 1** displays the picture of promoter hypermethylation of the TSHR, ATM, and P16 genes are generated by methylation-specific PCR (MSP). In PTC patients, 58.4% of the TSHR promoter methylation was detected compared to 41.6% in BTN patients, which was statistically significant (*P*=0.001). By contrast, PTC and BTN had no statistically significant difference in ATM methylation (*P*=0.873). In addition, P16 methylations were observed in 26.2% (17 of 61) of PTC patients and 73.8% (48 of 70) of BTN patients (*P*<0.001) (**Table S4**).

### 3.4 Association of clinicopathological characteristics and OCP levels with tumor-suppressor genes methylation

In two groups of PTC and BTN, excluding ATM, all promoter hypermethylation of selected genes was substantially different. Multiple logistic regression models were performed for OCP adjustment sensitivity examination (**Table 1**). Substantial association of TSHR and P16 methylation with a high risk of PTC and BTN has been shown as Model I (crude model). As compared to model I (crude model), chances of getting PTC and BTN from TSHR and P16 methylation raised in model II, III, IV, and V (total HCH, total DDE, total DDT, and total OCP adjusted models, respectively). Pesticide exposure exhibited a clear correlation with methylation of TSHR (OR = 3.98, 95% CI= 1.80-8.78, P=0.001) and P16 (OR = 5.65, 95% CI= 2.64-12.07, P<0.001) as opposed to the crude models.

**Table 1:** Evaluation of the effects of OCPs on gene promoter methylation using the logistic regression analysis.

Gene	Model	OR (95% CI)	*P*-value
TSHR^a^	**Model I**	3.34 (1.59-6.99)	**0.001**
	**Model II**	3.49 (1.64-7.41)	**0.001**
	**Model III**	3.90 (1.78-8.54)	**0.001**
	**Model IV**	4.27 (1.89-9.63)	**<0.001**
	**Model V**	3.98 (1.80-8.78)	**0.001**
ATM^a^	**Model I**	0.93 (0.38-2.22)	0.873
P16^b^	**Model I**	0.17 (0.08-0.37)	**<0.001**
	**Model II**	5.64 (2.65-12.00)	**<0.001**
	**Model III**	5.69 (2.66-12.16)	**<0.001**
	**Model IV**	5.81 (2.69-12.54)	**<0.001**
	**Model V**	5.65 (2.64-12.07)	**<0.001**

Model I is crude model, Model II is total HCH adjusted model, Model III is total DDE adjusted model, Model IV is total DDT adjusted model, Model V is total OCP adjusted model.

a Investigation of OCPs impacts in PTC subjects.

b Investigation of OCPs impacts in BTN subjects. *P*-values marked with bold indicate statistically significant *P*-values.

Based on logistic regression analysis, there was a positive relationship between OCP levels and DNA methylation (**Table S5**). Potential confounders have also been adjusted for age, smoking, gender, and exposure to pesticides. TSHR methylation was associated with γ-HCH (OR = 1.38, 95% CI: 1.09-1.76, P=0.007), 2,4-DDE (OR = 1.15, 95% CI: 1.05-1.25, P=0.001), 2, 4-DDT (OR = 1.49, 95% CI: 1.05-2.11, P=0.02), and 4, 4-DDT (OR = 1.23, 95% CI: 1.11-1.37, P<0.001). ATM methylation was related to 2,4-DDE (OR = 1.13, 95% CI: 1.01-1.27, P=0.03) and 4, 4-DDT (OR = 1.14, 95% CI: 1.01-1.30, P=0.03). P16 methylation was correlated with 4, 4-DDT (OR = 1.10, 95% CI: 1.00-1.20, P=0.03). By multivariable adjustment, 2,4-DDE effect was significantly attenuated on methylation of TSHR (OR = 1.01, 95% CI: 1.00-1.01, P=0.002), ATM (OR = 1.009, 95% CI: 1.00-1.017, P=0.04), and P16 (OR = 1.005, 95% CI: 1.00-1.009, P=0.03). Also, confounding variables significantly decreased the effect of 4, 4-DDT on methylation of TSHR (OR = 1.01, 95% CI: 1.00-1.02, P<0.001), ATM (OR = 1.009, 95% CI: 1.00-1.019, P=0.04), and P16 (OR = 1.008, 95% CI: 1.00-1.01, P=0.013). Moreover, after adjustment, TSHR methylation levels arising from γ-HCH (OR = 1.02, 95% CI: 1.00-1.03, P=0.016) and 2, 4-DDT (OR = 1.03, 95% CI: 1.00-1.05, P=0.014) exposure were reduced.

**Table S6** exhibits an association of the clinical and pathological factors and the gene methylation status. The most the ruptured capsule was seen in methylated TSHR status (66.7%, *P*<0.001). In methylated TSHR and unmethylated P16 status, the extracapsular nodal extension was 89.7% (*P*=0.014) and 93.2% (*P*<0.001), respectively. In PTC patients for organ invasion, there was a significant difference in the TSHR and ATM promoter methylation, and around 56.4% (*P*<0.001) and 44.9% (*P*=0.019) of patients showed methylated promoter, respectively. The most ATM methylation was found in-depth invasion >8 mm (42.9%, *P*=0.002). Also, in middle-sized tumors 2<size<4 cm, extreme methylation of TSHR was detected (43.6%, *P*=0.010). The promoter of TSHR and ATM were highly methylated in well-differentiated tumors in comparison with moderately-differentiated ones (76.9%, *P*=0.005 and 71.4%, *P*=0.014, respectively). The greatest number of nodes sampled (>5) were observed in TSHR (64.1%, *P*=0.002) and ATM (57.1%, *P*=0.012) methylation status.

**Figure 2:**
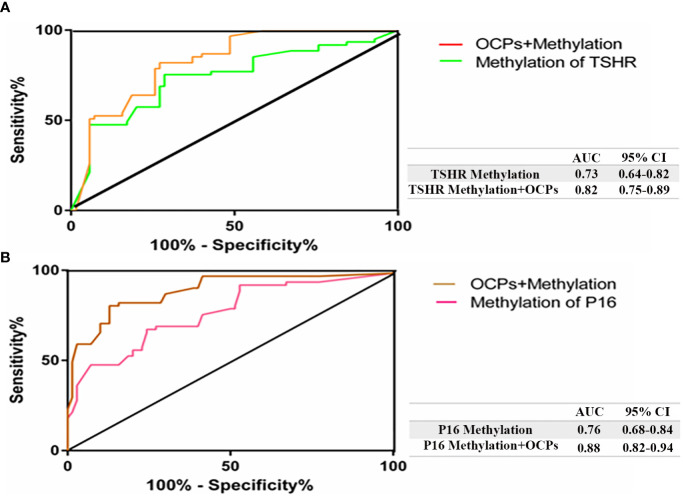
Receiver operating characteristic (ROC) curve confirmed the ability of OCPs to differentiate PTC from BTN. The diagonal line on the graph going from the lower left‐hand corner (0, 0) to the upper right‐hand corner (1, 1) serves as a reference line. Higher risk with thyroid tumors would yield a ‘curve’ that coincided with the left and top sides of the plot. **(A)** AUC showed higher value after the combination of TSHR methylation and OCPs exposure (orange) in PTC patients (0.82); **(B)** AUC demonstrated higher value after the combination of P16 methylation and OCPs exposure (brown) (0.88) in BTN patients. AUC, area under the curve; CsI, confidence interval.

The ROC curve analysis was utilized to discriminate PTC from BTN. One of the possible causes for TSHR methylation may be attribute to OCPs exposure which consequently leads to increase in the AUC levels to 0.82 with a 95% confidence interval (CI) ranging from 0.75 to 0.89, a sensitivity of 81.97% and a specificity of 72.86% (**Figure 2A**). OCP exposure along with P16 methylation had a higher AUC of 0.88 than just P16 methylation, with a 95% CI ranging from 0.82 to 0.94, a sensitivity of 80.33% and a specificity of 87.14% (**Figure 2B**).

### 3.5 Four histone modifications status in patients with thyroid tumors

**Figure 3:**
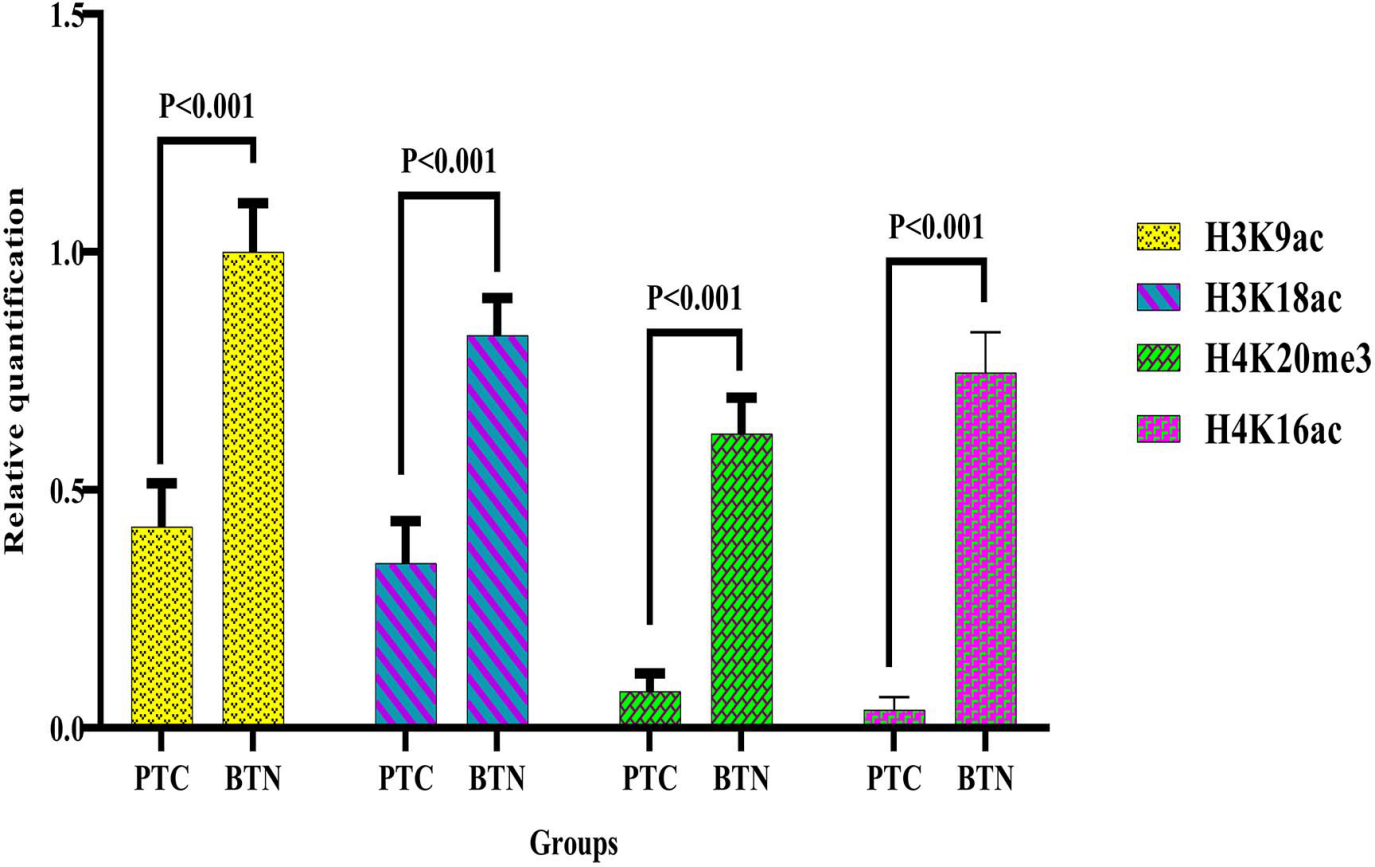
Comparison of histone modifications levels in PTC and BTN subjects. Significant differences were observed in four histone modifications between PTC and BTN patients (P <0.001). P values < 0.05 were considered statistically significant. PTC, Papillary thyroid carcinoma; BTN, Benign thyroid nodules.

In particular, H3K18ac, H3K9ac, H4K16ac, and H4K20me3 were selected for epigenetic modifications investigation due to their use in prior researches (7, 26, 43). Four different antibodies were utilized for the assessment of histone acetylation and methylation levels in thyroid tumors: H3K18ac, the identification of the acetylated lysine 18 in H3 histone; H3K9ac, the determination of the acetylated lysine 9 in H3 histone; H4K16ac, the acetylated lysine 16 in H4 histone recognition; H4K20me3, recognizing the tri-methylated lysine 20 in H4 histone. Compared with BTN patients, the levels of H3K18ac, H3K9ac, H4K16ac, and H4K20me3 marks were significantly lower in PTC patients (*P <*0.001) (**Figures 3**, **S1**, **Table S7**).

### 3.6 Association of clinicopathological characteristics and histone modifications

**Table S8** provides a relationship between clinicopathological information and histone modification. The ruptured capsule had a reverse correlation with H3K18ac (*P*=0.029) and H4K20me3 (*P*=0.014) and a direct relation to H3K9ac (*P*=0.044) levels. The levels of H3K9ac, H3K18ac, and H4K20me3 had an opposite association with extracapsular nodal extension (*P*<0.001, *P*=0.019, and *P*<0.001, respectively). Also, there was an adverse relationship between organ invasion and the level of H3K18ac (*P*=0.026) and H4K20me3 (*P*=0.015). In addition, depth invasion and tumor size had an inverse relation with H3K9ac, H3K18ac, and H4K20me3 (*P*<0.05).

### 3.7 Association of OCPs and histone modifications

Several logistic regression models were carried out to examine sensitivity adjusting for OCPs (**Table 2**). Model I (crude model) represent a significant relationship between the levels of H3K9 and H4K16 acetylation and PTC risk. Models II, III, IV, and V (total HCH, total DDE, total DDT, and total OCP adjusted models, respectively) demonstrate the reduction of chances of getting PTC from H3K9ac (OR = 3.68, 95% CI= 1.84-7.37, P<0.001) and H4K16ac (OR = 6.03, 95% CI= 2.32-15.66, *P*<0.001).

**Table 2:** Evaluation of the effects of OCPs on four histone modifications using the logistic regression analysis.

Histone modifications	Model	OR (CI-95%)	*P*-value
H3K9ac	**Model I**	3.71 (1.86-7.39)	**<0.001**
	**Model II**	3.67 (1.84-7.31)	**<0.001**
	**Model III**	3.69 (1.84-7.37)	**<0.001**
	**Model IV**	3.78 (1.87-7.66)	**<0.001**
	**Model V**	3.68 (1.84-7.37)	**<0.001**
H4K16ac	**Model I**	6.06 (2.33-15.75)	**<0.001**
	**Model II**	6.12 (2.36-15.90)	**<0.001**
	**Model III**	6.05 (2.32-15.72)	**<0.001**
	**Model IV**	6.10 (2.34-15.90)	**<0.001**
	**Model V**	6.03 (2.32-15.66)	**<0.001**

Model I is crude model, Model II is total HCH adjusted model, Model III is total DDE adjusted model, Model IV is total DDT adjusted model, Model V is total OCP adjusted model.

## 4 Discussion

Epigenetic control disruption contributes to irregular expression of genes, which can cause tumorigenesis and cancer progression (17). In reality, in thyroid tumors, tumor-suppressor and thyroid hormone-related differential genes expression are changed by mechanisms such as DNA cytosine residues methylation and the histone proteins posttranslational alterations; in turn, genes are selectively activated and or inactivated (44). This research was designed to examine the impacts of OCPs on the DNA methylation and histone alterations pattern in patients with thyroid tumors. The promoter hypermethylation of the TSHR gene in PTC, as opposed to BTN, was indicated in the current research. In addition, the logistic regression test suggested that OCPs could contribute to tumor-suppressors genes hypermethylation and, therefore may cause progression towards thyroid tumors. Also, adjusting for age, smoking, gender, and exposure to pesticides reduced the odds ratio of DNA methylation. Furthermore, the H3K18ac, H3K9ac, H4K16ac, and H4K20me3 marks assessment revealed dramatically lower levels of histone acetylation and methylation in patients with PTC than those with BTN. The results of the ROC analysis confirmed that OCP exposure could affect DNA methylation.

Adequate intake of iodine and maintenance of regular activity of the thyroid gland depends on the TSHR gene, one of the particular genes in thyroid tissue. TSHR gene expression was decreased by its promoter methylation, which can disrupt the iodine-concentrating capacity and thyroid function (12). TSHR methylation evaluation in thyroid tumors has demonstrated the hypermethylation of TSHR promoter in 33% of thyroid cell cancers and 30% of adenoma cases (9). Present results indicated significantly higher levels of TSHR methylation in PTC patients (58.4%) in comparison to BTN (41.6%) patients. Aberrant TSHR gene methylation has been defined as a new factor that differentiates malignancy from benign adenoma (45). Also, TSHR methylation is associated with capsule rupture, extracapsular nodal extension, organ invasion, the middle-sized tumor (2<size<4 cm), well-differentiated carcinoma, and >5 nodes sampled. In this regard, a meta-analysis review showed the increased methylation of TSHR in patients with metastasis of the lymph node as compared to those without metastasis of the lymph node (17). Therefore, one of the risk factors for PTC occurrence and promotion could be the TSHR gene methylation (17). Our findings showed that the methylation rate of the TSHR gene in the PTC specimens was significantly higher after adjustment for OCPs. Furthermore, γ-HCH, 2,4-DDE, 2,4-DDT, and 4, 4-DDT impacts on TSHR methylation were significantly reduced by confounding agents. These data show that OCP exposure can epigenetically alter primary genes that regulate thyroid function. No report has been documented on this issue so far. Also, the ROC curve of OCPs along with TSHR methylation was capable of distinguishing PTC from BTN. It has been suggested that mitochondria-mediated cell death was brought on by heptachlor (OCP) (46). Accordingly, it could be concluded that OCPs may contribute to cancer by interfering with the balance of cell death. No functional studies were performed to state that exposure to OCP lead to increase TSHR in this research.

In contrast to BTN, the present research illustrated P16 promoter hypomethylation in PTC (26.2%). Prior studies on PTC patients recorded 27% and 41% frequencies for P16 methylation, suggesting that P16 methylation is an event that frequently occurs in the epigenome of PTC subjects and may cause tumor formation (15, 47). The P16 in unmethylated status had a significant relation to extracapsular nodal extension. Contrary to our observations, the investigation of the correlation between several genes’ methylation and clinicopathological data showed that P16 methylation could lead to tumor promotion in head and neck squamous carcinoma (48). The disparity in tissue nature might cause this difference. Also, OCPs increased the levels of P16 methylation in the BTN group but not in the PTC group. Increased methylation of the P16 in the BTN group may be due to more exposure to OCP among BTN subjects. 2,4-DDE and 4,4-DDT influence on P16 methylation were considerably reduced by the confounding agents. Furthermore, ROC analysis depicted that OCPs exposure and P16 methylation differentiate BTN from PTC better than just P16 methylation. Research by Abolhassani et al. has consistently shown that OCPs can induce the methylation of P16 promoter, decrease P16 gene expression, and eventually develop colorectal cancer (28).

One of the essential ATM protein functions is to stop the cell cycle, which causes a sufficient delay or triggers the DNA repair mechanisms that are dependent on ATM and finally could cause stabilization of the genome (18). In this research, no substantial difference was detected for ATM methylation between PTC and BTN groups. However, the higher methylation of ATM promoter has been reported in PTC than in benign controls (12). Present observations exhibited a robust association between ATM methylation and organ invasion, depth invasion (≥8 mm), well-differentiated cancer, and >5 sampled nodes. Consistent with these data, in hepatocellular carcinoma, Yan et al. did not find a correlation between ATM methylation and clinicopathological characteristics such as histological differentiation and tumor size (21). Besides, confounding variables reduced the chances of ATM methylation arising from 2,4-DDE and 4,4-DDT influence. Since the 2,4-DDE level was higher in BTN rather than PTC, increasing the ATM methylation in the BTN group is not surprising.

Using the western blot technique, we assessed the level of posttranslational modifications of different amino acids of histones. Among the least 18 specific posttranslational chemical alterations of histones, acetylation and methylation have been extensively studied (25). This research indicates the higher levels of acetylated H3 at residues K9 and K18, acetylated H4 at residues K16, and tri-methylated H4 at residue K20 in BTN contrasting with PTC. Similar to Puppin et al. findings, our results revealed a decline in acetylated H3K18 in thyroid tumors (7). A reversal relation between acetylated H3K18 levels and the recurrence of prostate cancer has also been reported (49). Furthermore, the same researchers noticed a direct association between acetylated H3K18 levels and clinical repercussions in patients with lung and kidney cancer (50). Indeed, cAMP-dependent CBP/p300 transcriptional cofactor, often causes H3K18 acetylation (7). Since TSH exerts its effect by increasing cAMP levels in cells, it is unsurprising that H3K18ac levels in BTN have risen compared with PTC. Also, in agreement with Puppin et al., who reported a rise in H3K9 acetylation in undifferentiated carcinomas, the higher levels of H3K9ac in BTN than PTC were identified in our research (7). Multiple cellular processes such as the formation of heterochromatin, regulation of transcriptional activity, DNA repair system, replication of DNA, chromosome density, and genome stability correlate with the methylation of H4K20 (51). As a prevalent symptom in human cancer cells, the overall reduction of H4K20me3 and H4K16ac has been recorded by Fraga et al. (52). Similarly, in malignant breast tumors, Paydar et al. discovered H4K20me3 downregulation (38). No functional investigations were carried out to prove that exposure to OCP increased histone acetylation in benign nodules in this area.

An inverse relationship between histone modifications and pathological characteristics such as ruptured capsule, extracapsular nodal extension, organ invasion, depth invasion, and tumor size was observed in this research. On the other hand, owing to total OCP exposure, decreased levels of acetylation of H3K9 and H4K16 were noticed. Therefore, it could be inferred that PTC progression may be due to reduced histone acetylation resulting from OCP exposure. Contrary to our observations, Song et al. showed increased core histones acetylation could arise from dieldrin exposure (27). Many variables, such as single or multiple pesticides, the duration of exposure, dosage, and type of pesticide may affect the results of various studies.

## 5 Conclusions

It seems that the risk of PTC is related to TSHR promoter hypermethylation, while PTC and BTN patients showed no significant difference for ATM. Also, OCPs can lead to the induction of TSHR and P16 promoter methylation. Moreover, total HCH and total DDE exposure resulted in reduced H3K9ac levels, and decreased H4K16ac levels were caused by total DDE exposure. Accordingly, due to the influence of OCPs on DNA methylation and histone modifications, we believe these pesticides might contribute to the occurrence of thyroid tumors through epigenetic alterations.

## Data availability statement

The original contributions presented in the study are included in the article/**Supplementary Material**. Further inquiries can be directed to the corresponding author.

## Ethics statement

The studies involving human participants were reviewed and approved by as a sign of gratitude for their cooperation, we would like to thank subjects participating in this study. The research was accepted by the ethics board of Kerman University of Medical Sciences (Code No.: IR. KMU. REC.1398.370). The patients/participants provided their written informed consent to participate in this study.

## Author contributions

GA conceived the study and designed the survey and provided continuous guidance throughout the study and interpreted the data. FS and MA-J collected samples. FS and MA performed all experiments, oversaw data collection and analysis, and drafted the manuscript. GA, HN, MRA and MS helped the survey and analyzed data. All authors contributed to the article and approved the submitted version.
