# Supplementary material for: Organochlorine pesticides and epigenetic alterations in thyroid tumors
Source: Front Endocrinol (Lausanne). 2023 Jul 25;14:1130794. doi: 10.3389/fendo.2023.1130794 (PMC10409498; doi:10.3389/fendo.2023.1130794)
Supplement: Supplementary file 1 [file DataSheet_1.docx]

***Supplementary Material***

**Organochlorine pesticides and epigenetic alterations in thyroid tumors**

| Table S1 | Primer sequences, annealing temperatures, and product sizes of specific gene used in MSP assays. |
| --- | --- |
| Table S2 | Comparison of clinical and pathological characteristics between PTC and BTN subjects. |
| Table S3 | The OCPs (ng/ml) levels in PTC and BTN. |
| Table S4 | Methylation status of promoter of TSHR, ATM and P16 genes in thyroid tumor patients. |
| Table S5 | Association between DNA methylation and OCPs. |
| Table S6 | Association of clinicopathological parameters and three tumor suppressor genes methylation status in PTC patients. |
| Figure S1 | Western blot of different modifications at H3K9ac, H3K18ac, H4K16ac, and H4K20me3 |
| Table S7 | Comparison of histone modifications in thyroid tumor patients. |
| Table S8 | Association of clinicopathological characteristics and histone modification in PTC patients. |

**Table S1.** Primer sequences, annealing temperatures, and product sizes of specific gene used in MSP assays.

| PCR cycles | The amount of DNA | Annealing temperature | Product  (bp) | Primer sequence | Gene name |
| --- | --- | --- | --- | --- | --- |
| 35 | 2 µl | 58 | 483 | 5ؘ-GGTTTTGGAGAGAATTAATGGGAG-3ؘ  5ؘ-AACTCTAAAATCCTCCTCCTAATAACAC-3ؘ | Stage I-PCR  TSHR-F  TSHR-R |
| 35  35 | 2 µl | 53  53 | 88  91 | 5ؘ-TGTAGAGTTGAGAATGAGGTGATTTC-3ؘ  5ؘ-CAACTACAACAAATCCGCCG-3ؘ  5ؘ-TGTAGAGTTGAGAATGAGGTGATTTT-3ؘ  5ؘ-CACCAACTACAACAAATCCACCA-3ؘ | Stage II-PCR  TSHR-MF  TSHR-MR  TSHR-UF  TSHR-UR |
| 35 | 2 µl | 58 | 182 | 5ؘ-TTTTTAGATTTGGAGGGG-3ؘ  5ؘ-CAAAACACTACCCCAAAACATT-3ؘ | Stage I-PCR  ATM-F  ATM-R |
| 35  35 | 2 µl | 66.5  62 | 178  179 | 5ؘ-GCGGGGAGGACGACGAGGGC-3ؘ  5ؘ-GCATCCAATATCACGCGATCTCCG-3ؘ  5ؘ-GTGGG GAGGATGATGAGGGT-3ؘ  5ؘ-CACATCCAATATCACACAATC TCCA-3ؘ | Stage II-PCR  ATM -MF  ATM -MR  ATM -UF  ATM -UR |
| 40 | 3 µl | 64 | 258 | 5՜-GAAGAAAGAGGAGGGGTTGG-3՜  5՜-CCACCTAAATCGACCTCCGACCG-3՜ | Stage I-PCR  P16-F  P16-R |
| 35  40 | 1.5 µl | 70  62 | 150  151 | 5՜-TTATTAGAGGGTGGGGCGGATCGC-3՜  5՜-GACCCCGAACCGCGACCGTAA-3՜  5՜-TTATTAGAGGGTGGGGTGGATTGT-3՜  5՜-CAACCCCAAACCACAACCATAA-3՜ | Stage II-PCR  P16-MF  P16-MR  P16-UF  P16-UR |

**Table S2.** Comparison of clinical and pathological characteristics between PTC and BTN subjects.

| **Parameter** | | **PTC** | **BTN** | ***P*-value** |
| --- | --- | --- | --- | --- |
| **Age (years)** (Mean ± SEM) **(n)** | | 40 ± 1.7 (61) | 47 ± 1.6 (70) | **0.005** |
| **Gender (male/female)** | | 15/46 (24.6/75.4) | 14/56(20/80) | 0.52 |
| **Smoking (yes/no) (n (%))** | | 2/59 (3.3/96.7) | 4/66 (5.7/94.3) | 0.35 |
| **Agriculture precedent (yes/no) (n (%))** | | 16/45 (26.2/73.8) | 9/61 (13/87) | 0.79 |
| **Nodule size (cm) (Mean ± SEM)** | | 2.3 ± 0.2 | None |  |
| **Depth of Stromal Invasion (mm) (Mean ± SEM)** | | 9.63 ± 1.6 | None |  |
| **Number of Nodes Sampled** | | 6 ± 0.67 | None |  |
| **Number of Positive Nods** | | 1.98 ± 0.5 | None |  |
| **Stage (n (%))** | **Stage 1** | 49 (80.3) | None |  |
|  | **Stage 2** | 6 (9.8) | None |  |
|  | **Stage 3** | 4 (6.6) | None |  |
|  | **Stage 4** | 1 (1.6) | None |  |
| **Histologic (n (%))** | **Well differentiated** | 37 (60.65) | None |  |
|  | **Moderately differentiated** | 24 (39.35) | None |  |
| **TNM (n (%))** | **T1** | 15 (24.6) | None |  |
|  | **T2** | 21 (34.4) | None |  |
|  | **T3** | 18 (29.5) | None |  |
|  | **T4** | 7 (11.5) | None |  |
|  | **N0** | 43 (70.5) | None |  |
|  | **N1** | 17 (27.9) | None |  |
|  | **Nx** | 1 (1.6) | None |  |
|  | **M0** | 0 | None |  |
|  | **M1** | 0 | None |  |
|  | **Mx** | 61 (100) | None |  |
| **Organ Invasion**  **(n (%))** | **Seen** | 21 (34.4) | None |  |
|  | **Not seen** | 38 (62.3) | None |  |
| **Extracapsular nodal extension**  **(n (%))** | **Yes** | 47 (77) | None |  |
|  | **No** | 12 (19.7) | None |  |
|  | **Unknown** | 2 (3.3) | None |  |
| **Ruptured Capsule**  **(n (%))** | **Yes** | 24 (39.3) | None |  |
|  | **No** | 35 (57.4) | None |  |
|  | **Unknown** | 2 (3.3) | None |  |

Data are expressed as numbers of individuals or means ± SEM and comparisons were made by the Chi-square test or Student’s-sample t-test, respectively. BTN: Benign thyroid nodule; PTC: Papillary thyroid carcinoma.

**Table S3.** The OCPs (ng/ml) levels in PTC and BTN (1).

| **Parameters** | **PTC (61)**  **Mean ± SEM** | **BTN (70)**  **Mean ± SEM** | ***P*-value** |
| --- | --- | --- | --- |
| **α-HCH (ng/ml)** | 0.78 ± 0.21 | 1.09 ± 0.27 | 0.371 |
| **β-HCH (ng/ml)** | 1.24 ± 0.21 | 1.62 ± 0.40 | 0.428 |
| **γ-HCH (ng/ml)** | 1.32 ± 0.30 | 1.10 ± 0.23 | 0.569 |
| **2,4-DDE (ng/ml)** | 5.64 ± 0.76 | 6.38 ± 0.82 | 0.517 |
| **4,4-DDE (ng/ml)** | 1.94 ± 0.39 | 1.80 ± 0.36 | 0.797 |
| **2,4-DDT (ng/ml)** | 1.32 ± 0.14 | 1.30 ± 0.12 | 0.931 |
| **4,4-DDT (ng/ml)** | 4.18 ± 0.51 | 4.55 ± 0.49 | 0.610 |

α-HCH: α–Hexachlorocyclohexane; β-HCH: β-Hexachlorocyclohexane; γ-HCH: γ-Hexachlorocyclohexane; 2,4-DDE: 2,4-Dichlorodiphenyldichloroethylene; 4,4-DDE: 4,4-Dichlorodiphenyldichloroethylene; 2,4-DDT: 2,4-Dichlorodiphenyltrichloroethane; 4,4-DDT: 4,4-Dichlorodiphenyltrichloroethane.

**Table S4.** Methylation status of promoter of TSHR, ATM and P16 genes in thyroid tumor patients.

| Gene | Group | Methylated (n (%)) | Unmethylated (n (%)) | P value |
| --- | --- | --- | --- | --- |
| TSHR | PTC | 45 (58.4) | 16 (29.6) | **0.001** |
|  | BTN | 32 (41.6) | 38 (70.4) |  |
| ATM | PTC | 49 (46.2) | 12 (48) | 0.873 |
|  | BTN | 57 (53.8) | 13 (52) |  |
| P16 | PTC | 17 (26.2) | 44 (66.7) | **<0.001** |
|  | BTN | 48 (73.8) | 22 (33.3) |  |

**Table S5.** Association between DNA methylation and OCPs.

| Adjusted | | | Crude | | | Parameters |
| --- | --- | --- | --- | --- | --- | --- |
| *P*-value | CI-95% | OR | *P*-value | CI-95% | OR |  |
| 0.18  0.26  0.83 | 0.97-1.00  0.97-1.00  0.98-1.01 | 0.99  0.99  0.99 | 0.33  0.25  0.48 | 0.77-1.09  0.74-1.07  0.78-1.12 | 0.917  0.89  0.94 | α-HCH  TSHR  ATM  P16 |
| 0.34  0.98  0.47 | 0.99-1.01  0.98-1.01  0.99-1.01 | 1.005  1.00  1.004 | 0.45  0.92  0.58 | 0.91-1.21  0.84-1.15  0.91-1.17 | 1.05  0.99  1.03 | β-HCH  TSHR  ATM  P16 |
| **0.016**  1  0.33 | 1.00-1.03  0.99-1.04  0.99-1.01 | 1.02  1.02  1.006 | **0.007**  0.09  0.62 | 1.09-1.76  0.95-1.84  0.88-1.21 | 1.38  1.32  1.04 | γ-HCH  TSHR  ATM  P16 |
| **0.002**  **0.04**  **0.03** | 1.00-1.01  1.00-1.017  1.00-1.009 | 1.01  1.009  1.005 | **0.001**  **0.03**  0.08 | 1.05-1.25  1.01-1.27  0.99-1.11 | 1.15  1.13  1.05 | 2,4-DDE  TSHR  ATM  P16 |
| 0.33  0.48  0.39 | 0.99-1.01  0.99-1.01  0.99-1.01 | 1.00  1.004  1.004 | 0.22  0.48  0.88 | 0.95-1.22  0.89-1.25  0.90-1.12 | 1.08  1.06  1.009 | 4,4-DDE  TSHR  ATM  P16 |
| **0.014**  0.57  0.17 | 1.00-1.05  0.97-1.03  0.99-1.03 | 1.03  1.008  1.01 | **0.02**  0.5  0.57 | 1.05-2.11  0.76-1.73  0.79-1.50 | 1.49  1.15  1.09 | 2,4-DDT  TSHR  ATM  P16 |
| **<0.001**  **0.04**  **0.013** | 1.00-1.02  1.00-1.019  1.00-1.01 | 1.01  1.009  1.008 | **<0.001**  **0.03**  **0.03** | 1.11-1.37  1.01-1.30  1.00-1.20 | 1.23  1.14  1.10 | 4,4-DDT  TSHR  ATM  P16 |

The estimation of the associations between DNA methylation and OCPs was performed by the continuous logistic regression model. ^a^ Adjusted for age, smoking, gender, and exposure.

**Table S6.** Association of clinicopathological parameters and three tumor suppressor genes methylation status in PTC patients.

| Characteristics | | TSHR n (%) | | | ATM n (%) | | | P16 n (%) | | |
| --- | --- | --- | --- | --- | --- | --- | --- | --- | --- | --- |
|  |  | Methylated | Unmethylated | P value | Methylated | Unmethylated | P value | Methylated | Unmethylated | P value |
| Ruptured Capsule | Yes | 26 (66.7) | 3 (13.6) | **<0.001** | 26 (53.1) | 3 (25) | 0.08 | 6 (35.3) | 23 (52.3) | 0.23 |
|  | No | 13 (33.3) | 19 (86.4) |  | 23 (46.9) | 9 (75) |  | 11 (64.7) | 21 (47.7) |  |
| Extracapsular nodal extension | Yes | 35 (89.7) | 14 (63.6) | **0.014** | 40 (81.6) | 9 (75) | 0.60 | 8 (47.1) | 41 (93.2) | **<0.001** |
|  | No | 4 (10.3) | 8 (36.4) |  | 9 (18.4) | 3 (25) |  | 9 (52.9) | 3 (6.8) |  |
| Organ Invasion | Seen | 22 (56.4) | 1 (4.5) | **<0.001** | 22 (44.9) | 1 (8.3) | **0.019** | 9 (52.9) | 14 (31.8) | 0.12 |
|  | Not Seen | 17 (43.6) | 21 (95.5) |  | 27 (55.1) | 11 (91.7) |  | 8 (47.1) | 30 (68.2) |  |
| Depth invasion (mm) | < 4 | 7(17.9) | 9(40.9) | 0.13 | 8(16.3) | 8(66.7) | **0.002** | 3(17.6) | 13(29.5) | 0.47 |
|  | 4<x<8 | 15(38.5) | 7(31.8) |  | 20(40.8) | 2(16.7) |  | 8(47.1) | 14(31.8) |  |
|  | > 8 | 17(43.6) | 6(27.3) |  | 21(42.9) | 2(16.7) |  | 6(35.3) | 17(38.6) |  |
| Tumor size (cm) | < 2 | 9(23.1) | 12(54.5) | **0.010** | 18(36.7) | 3(25) | 0.58 | 6(35.3) | 15(34.1) | 0.69 |
|  | 2<x<4 | 17(43.6) | 9(40.9) |  | 21(42.9) | 5(41.7) |  | 6(35.3) | 20(45.5) |  |
|  | > 4 | 13(33.3) | 1(4.5) |  | 10(20.4) | 4(33.3) |  | 5(29.4) | 9(20.5) |  |
| Histologic | Well differentiated | 30(76.9) | 9(40.9) | **0.005** | 35(71.4) | 4(3.3) | **0.014** | 11(64.7) | 28(63.6) | 0.93 |
|  | Moderately differentiated | 9(23.1) | 13(59.1) |  | 14(28.6) | 8(66.7) |  | 6(35.3) | 16(36.4) |  |
| No. of nodes sampled | <5 | 14(35.9) | 17(77.3) | **0.002** | 21(42.9) | 10(83.3) | **0.012** | 7(41.2) | 24(54.5) | 0.34 |
|  | >5 | 25(64.1) | 5(22.7) |  | 28(57.1) | 2(16.7) |  | 10(58.8) | 20(45.5) |  |

**
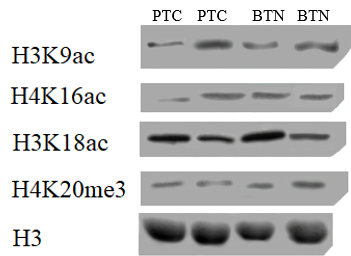
**

**Figure S1.** Western blot of histone modifications in different patients (number 1 to 4) with thyroid tumors. Total Histone H3 was as a loading control.

**Table S7.** Comparison of histone modifications in thyroid tumor patients.

| Histone modifications | Group (n) | Mean ± SEM | P value |
| --- | --- | --- | --- |
| H3K9ac | PTC (61) | 0.36 ± 0.08 | **<0.001** |
|  | BTN (70) | 0.90 ± 0.09 |  |
| H4K16ac | PTC (61) | 0.25 ± 0.02 | **<0.001** |
|  | BTN (70) | 0.67 ± 0.08 |  |
| H3K18ac | PTC (61) | 0.29 ± 0.07 | **<0.001** |
|  | BTN (70) | 0.74 ± 0.07 |  |
| H4K20me3 | PTC (61) | 0.19 ± 0.03 | **<0.001** |
|  | BTN (70) | 0.55 ± 0.07 |  |

**Table S8.** Association of clinicopathological characteristics and histone modification in PTC patients.

| Characteristics | | H3K9ac | | H4K16ac | | H3K18ac | | H4K20me3 | |
| --- | --- | --- | --- | --- | --- | --- | --- | --- | --- |
|  |  | Mean ± SEM | P value | Mean ± SEM | P value | Mean ± SEM | P value | Mean ± SEM | P value |
| Ruptured Capsule | Yes | 0.17 ± 0.09 | **0.044** | 0.023 ± 0.02 | 0.179 | 0.10 ± 0.06 | **0.029** | 0.08 ± 0.03 | **0.014** |
|  | No | 0.049 ± 0.11 |  | 0.30 ± 0.04 |  | 0.43 ± 0.12 |  | 0.27 ± 0.06 |  |
| Extracapsular nodal extension | Yes | 0.20 ± 0.06 | **<0.001** | 0.25 ± 0.02 | 0.125 | 0.20 ± 0.07 | **0.019** | 0.12 ± 0.03 | **<0.001** |
|  | No | 1.00 ± 0.25 |  | 0.35 ± 0.09 |  | 0.65 ± 0.20 |  | 0.48 ± 0.13 |  |
| Organ Invasion | Seen | 0.24 ± 0.11 | 0.25 | 0.23 ± 0.02 | 0.16 | 0.10 ± 0.06 | **0.026** | 0.08 ± 0.03 | **0.015** |
|  | Not seen | 0.43 ± 0.10 |  | 0.30 ± 0.04 |  | 0.41 ± 0.11 |  | 0.26 ± 0.05 |  |
| Depth invasion | < 4 | 0.77 ± 0.20 | **0.001** | 0.33 ± 0.06 | 0.353 | 0.61 ± 0.18 | **0.004** | 0.38 ± 0.09 | **0.001** |
|  | 4<x<8 | 0.35 ± 0.11 |  | 0.24 ± 0.03 |  | 0.33 ± 0.13 |  | 0.20 ± 0.06 |  |
|  | > 8 | 0.04 ± 0.03 |  | 0.25 ±0.03 |  | 0.0 ± 0.0 |  | 0.03 ±0.01 |  |
| Tumor size | < 2 | 0.53 ± 0.14 | **0.043** | 0.31 ± 0.05 | 0.399 | 0.52 ± 0.15 | **0.034** | 0.33 ± 0.08 | **0.004** |
|  | 2<x<4 | 0.38 ± 0.12 |  | 0.27 ± 0.03 |  | 0.23 ± 0.11 |  | 0.15 ± 0.04 |  |
|  | > 4 | 0.00 ± 0.00 |  | 0.21 ± 0.02 |  | 0.00 ± 0.00 |  | 0.00 ± 0.00 |  |

1. Salimi F, Asadikaram G, Abolhassani M, Pourfarjam Y, Nejad HZ, Abbasi-Jorjandi M, et al. Organochlorine pesticides induce thyroid tumors through oxidative stress; an in vivo and in silico study. Environmental Science and Pollution Research. 2023;30(15):45046-66.
